# Tethering (Arene)Ru(II) Acylpyrazolones Decorated with Long Aliphatic Chains to Polystyrene Surfaces Provides Potent Antibacterial Plastics

**DOI:** 10.3390/ma13030526

**Published:** 2020-01-22

**Authors:** Corrado Di Nicola, Fabio Marchetti, Riccardo Pettinari, Alessia Tombesi, Claudio Pettinari, Iolanda Grappasonni, Paul J. Dyson, Stefania Scuri

**Affiliations:** 1 School of Science and Technology, Chemistry Section, University of Camerino, Via S. Agostino 1, 62032 Camerino Macerata, Italy; fabio.marchetti@unicam.it (F.M.); alessia.tombesi@unicam.it (A.T.); 2 School of Pharmacy, Chemistry Section, University of Camerino, Via S. Agostino 1, 62032 Camerino Macerata, Italy; riccardo.pettinari@unicam.it (R.P.); claudio.pettinari@unicam.it (C.P.); iolanda.grappasonni@unicam.it (I.G.); 3 Institut des Sciences et Ingénierie Chimiques, École Polytechnique Fédérale de Lausanne (EPFL), 1015 Lausanne, Switzerland; paul.dyson@epfl.ch

**Keywords:** bioorganometallic chemistry, Ruthenium(II) complexes, acylpyrazolones, nanoformulation, tethering on polyethylene and polystyrene, antibacterial composite materials

## Abstract

The acylpyrazolone proligands HQ^R^ (HQ^R^ in general, in detail: HQ^Cy^ = 1-phenyl-3-methyl-4-carbonylcyclohexyl-5-pyrazolone, 4-C(O)-phenyl, HQ^Ph^ = 1-phenyl-3-methyl-4-benzoyl-5-pyrazolone, HQ^C17^ = 1-phenyl-3-methyl-4-stearoyl-5-pyrazolone, HQ^C17,Ph^ = 1-phenyl-3-stearyl-4-benzoyl-5-pyrazolone) were synthesized and reacted with (arene)Ru(II) acceptors affording complexes [(arene)Ru(Q^R^)Cl] (arene = cymene (cym) or hexamethylbenzene (hmb)). The complexes were characterized by elemental analyses, thermogravimetric analysis-Differntial Thermal Analysis (TGA-DTA), IR spectroscopy, ESI-MS and ^1^H, and ^13^C NMR spectroscopy. Complexes [(arene)Ru(Q^R^)Cl] where Q^R^ = Q^C17^ and Q^C17,Ph^, due to the long aliphatic chain in the ligand, afford nanometric dispersions in methanol via self-assembly into micellar aggregates of dimensions 50–200 nm. The antibacterial activity of the complexes was established against *Escherichia coli* and *Staphylococcus aureus*, those containing the ligands with a long aliphatic chain being the most effective. The complexes were immobilized on polystyrene by a simple procedure, and the resulting composite materials showed to be very effective against *E. coli* and *S. aureus*.

## 1. Introduction

**Chart 1:**
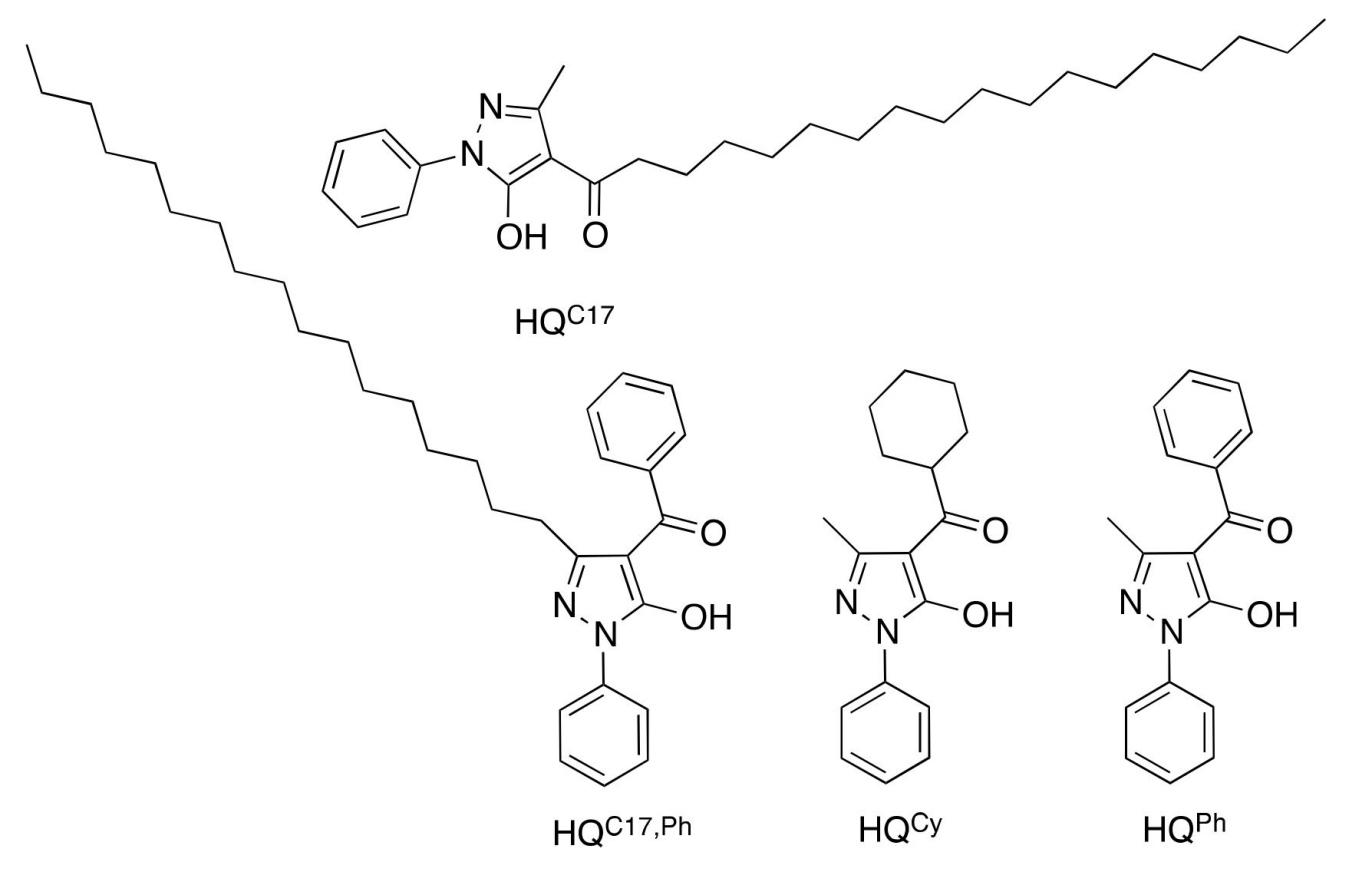
HQ^R^ proligands used in this work.

A wide range of ruthenium-based complexes display promising properties for cancer chemotherapy and have emerged as a potential alternative to the platinum-derived therapeutics [1,2,3]. In recent years Ru(III) and Ru(II) have successfully entered clinical trials [4,5,6,7,8,9,10] and their mechanisms of anticancer action have been investigated [11,12,13]. However, ruthenium complexes possess additional interesting medicinal properties, such as antiparasitic [14] and antibacterial [15,16,17] activity. In this respect, a general approach based on tethering antibacterial agents such as deferoxamine B to (arene)Ru(II)−platforms has been proposed [18]. The incorporation of antimicrobials within plastic appliances and surfaces has attracted over the last few years a great deal of research and development attention because of widespread concerns at all levels in safety and hygiene [19]. The types of polymeric systems with antimicrobial activity can be divided into four main groups: (a) polymers with intrinsic antimicrobial activity; (b) polymers that are chemically or physically modified to covalently incorporate an antimicrobial function; (c) polymers containing organic antimicrobial substances non covalently linked to the matrix; (d) polymers containing inorganic antimicrobial substances non covalently linked to the matrix [20]. The current industrial production of antimicrobial plastics is mainly based on the use of silver-based antimicrobial additives, especially silver nanoparticles. However, recent studies seem to indicate a potential toxicity to humans deriving from the use and massive release of metal nanoparticles into the environment, and alternative technological solutions to the use of nanoparticles are the subject of numerous studies [21,22]. Our interests are devoted to the development of sustainable methodologies that afford scalable functional materials endowed with antimicrobial activity [23,24,25]. We have recently reported the preparation of composite materials formed by loading (cymene)Ru(II) curcumin complexes, previously reported to be potent antitumor agents [26], on porous carbon adsorbents, which display efficient antibacterial activity [27]. These features are relevant to the use of such carbon composites in air filters for some specific applications in the food and pharmaceutical industries, such as in air conditioning equipment, especially for hospitals, in operating rooms, [28] etc. Previously we reported several (arene)Ru(II) complexes [29,30,31,32] with acylpyrazolone ligands, which are β-diketones with a pyrazole fused to the chelating arms, and they exhibit anticancer activity [33,34,35]. The combination of both the Q^C17^ (HQ^C17^ = 1-phenyl-3-methyl-4-stearoyl-5-pyrazolone, see Chart 1) and PTA (PTA = 1,3,5-Triaza-7-phosphaadamantane) ligands on the same (arene)Ru(II) scaffold resulted in highly cytotoxic compounds with a potency comparable to cisplatin in human ovarian A2780 cancer cells, indeed being equally cytotoxic to both the A2780 and the cisplatin-resistant A2780cisR cancer cell lines and similar to other (arene)Ru(II) complexes bearing both a PTA ligand and a long hydrophobic chain [36]. Due to their tendency to release the Q^C17^ and form the same species as RAPTA-C inside a cell, their strong cytotoxicity is particularly interesting, as they can be considered as prodrugs able to generate RAPTA-C, which is essentially not cytotoxic, in part due to limited cellular uptake [37].

Herein, we extend our nascent studies to the synthesis of a new proligand HQC17,Ph bearing a long aliphatic chain in the three-position of the pyrazole, usually occupied by a methyl group (Chart 1) and the corresponding (arene)Ru(II) derivatives (arene = cymene and hexamethylbenzene). All substances were subjected to microbiological tests against *Escherichia coli* and *Staphylococcus aureus*. For comparative purposes, the previously reported (arene)Ru(II) complexes with other QR ligands (Chart 1) were also screened against these organisms. Additionally, complexes containing ligands Q^C17^ and Q^Ph,C17^ showed amphipathic behavior due to the long hydrophobic chain present in their structures. this structural feature was exploited to immobilize the complexes onto a polystyrene surface using a simple procedure, affording antibacterial composite plastics.

Polystyrene was chosen in this study as it is a well-known thermoplastic polymer, the scale of its production being several million tonnes per year, which can be solid or foamed, widely used for many applications including protective packaging, bottles, trays, tumblers, disposable cutlery, containers, lids, etc. etc. due to its flexibility, easy processability, thermal stability, environmental recyclability, and inexpensive properties [38]. In consideration of this variety of possible applications, polystyrene is a suitable polymer to be tested for the preparation of new antimicrobial composite materials through a surface treatment aimed at anchoring antimicrobial additives. Additionally, polystyrene presents a good solubility in some organic solvents, such as acetone which was advantageously used in the preparation of our composite materials.

## 2. Results

### 2.1. Synthesis and Characterization of the HQ^R^ Proligands

**Scheme 1:**
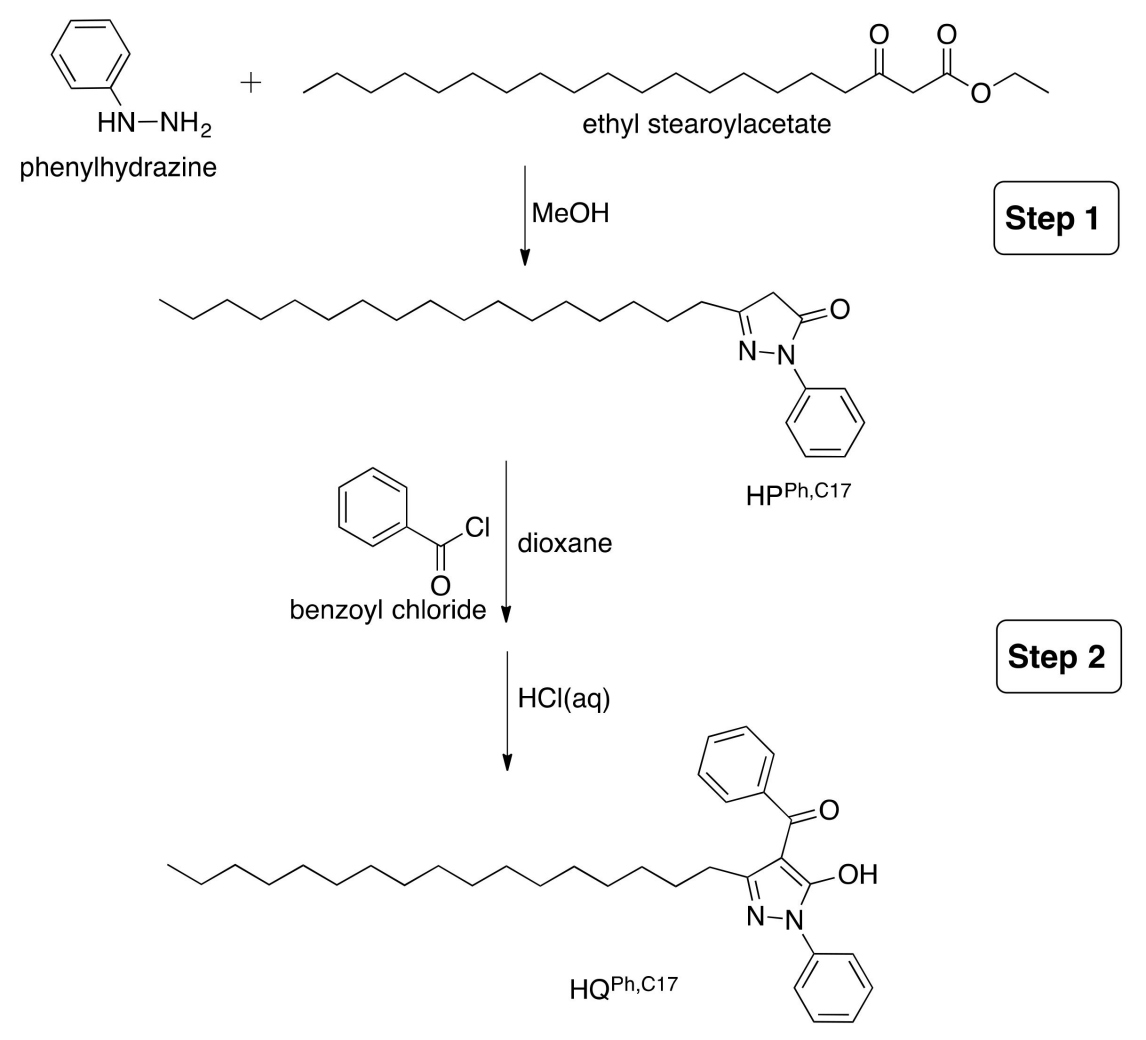
Synthetic procedure for proligand HQ^Ph,C17^.

Proligands HQ^Ph^, HQ^Cy,^ and HQ^C17^ were prepared following a published procedure [34]. Proligand HQ^Ph,C17^ was synthesized for the first time following a two-step procedure, by preparing the precursor 3-heptadecyl-1-phenyl-1*H*-pyrazol-5(4*H*)-one (HP^Ph,C17^ in Scheme 1) through the condensation of phenylhydrazine and ethyl stearoylacetate in methanol. The precursor was then acylated at the C-4 position of the pyrazole ring by reaction with benzoyl chloride in basic (calcium hydroxide) [39] dioxane at reflux. Subsequent acidification afforded the proligand (HQ^Ph,C17^ in Scheme 1) in high yield as a pale pink solid powder that is insoluble in water. 

The HQ^Ph,C17^ proligand is a low melting solid, soluble in chlorinated solvents, acetone, DMF, diethyl ether and n-hexane, and slightly soluble in hot ethanol. The broadband between 2500 and 2800 cm^−1^ in the IR spectrum indicates the prevalence of the keto-enol tautomeric form in the solid-state, with strong adsorptions at 2921 and 2852 cm^−1^ due to the long aliphatic chain at C3 position of pyrazolone ring and at 1599 and 1546 cm^−1^ due to ν(C=O) stretching modes. The ^1^H and ^13^C NMR spectra recorded in CDCl_3_ display all the expected proton and carbon resonances for the phenyl rings and those of the aliphatic chain in position 3 of the pyrazolone ring.

### 2.2. Synthesis and Characterization Complexes 1–8

**Scheme 2:**
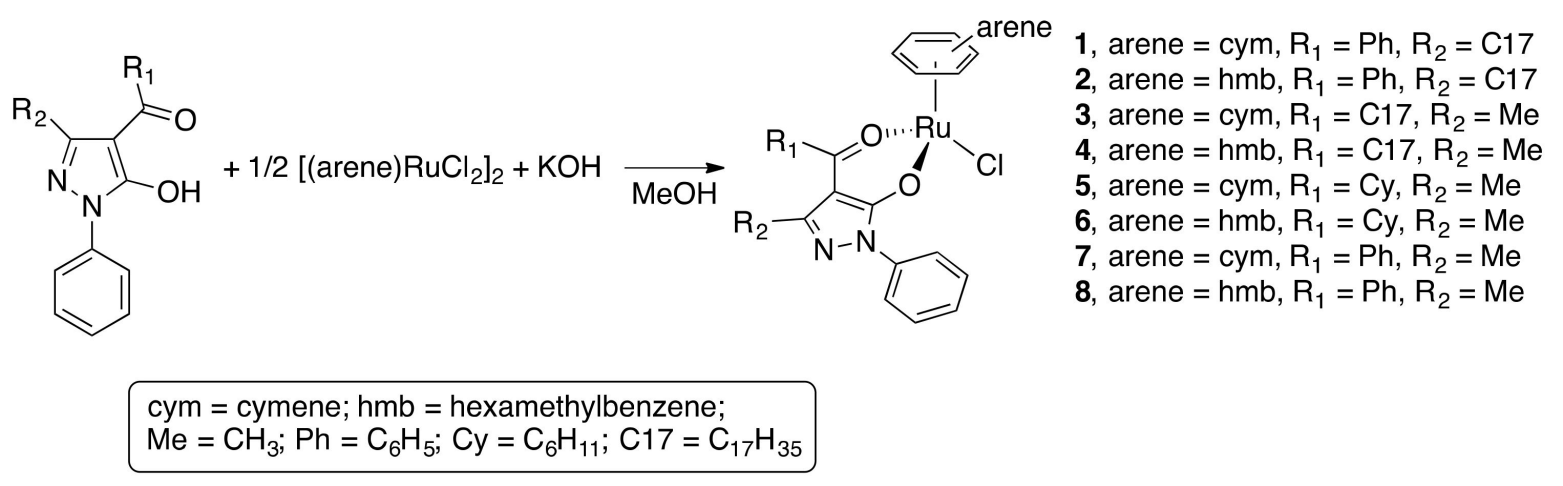
Synthetic procedure for complexes 1–8.

Complexes 1–8 were prepared by the reaction of proligands HQ^R^ with the appropriate [(arene)RuCl_2_]_2_ dimers (where arene is cymene (cym) or hexamethylbenzene (hmb)) in methanol in the presence of KOH (Scheme 2). 

Complexes 3–8 were previously reported and characterized [31,32], whereas 1 and 2 are new. Complex 2 is a low melting solid, whereas 1 is a viscous liquid. Both are highly soluble in alcohols, acetone, acetonitrile, DMSO, DMF, diethyl ether, *n*-hexane, and chlorinated solvents. The IR spectra of 1 and 2 display a typical shift of the ν(C=O) stretches, with respect to that observed for the free neutral ligand, in accordance with the coordination of the O_2_-chelating moiety to the ruthenium(II) centers. Additional bands in the far-IR region appear upon coordination, and those at around 450 and 280 cm^−1^ were assigned to ν(Ru-O), and ν(R-Cl) stretches (Figure S1 in Supplementary) [29,31,32,40]. The ^1^H and ^13^C NMR spectra of 1 and 2 were recorded in CDCl_3_ and display a set of signals due to the Q^Ph,C17^ ligand, slightly shifted in frequency in comparison with the free proligand (Figures S2–S7 in Supplementary), the typical AA’XX’ pattern of the cymene protons in 1 and a unique resonance for the CH_3_ protons in hexamethylbenzene in 2. The proton and carbon assignments were made on the basis of previous studies on analogous complexes [29,31,32,40]. The positive ESI-MS spectra of 1 and 2 show a peak corresponding to the [(arene)Ru(Q^Ph,C17^)]^+^ ion arising from the detachment of chloride. Both complexes undergo progressive decomposition from 180 to 850 °C, as found by thermogravimetric analysis (TGA) analysis.

### 2.3. Microbiological Study on Complexes 1–8 and Proligands HQ^R^

**Figure 1:**
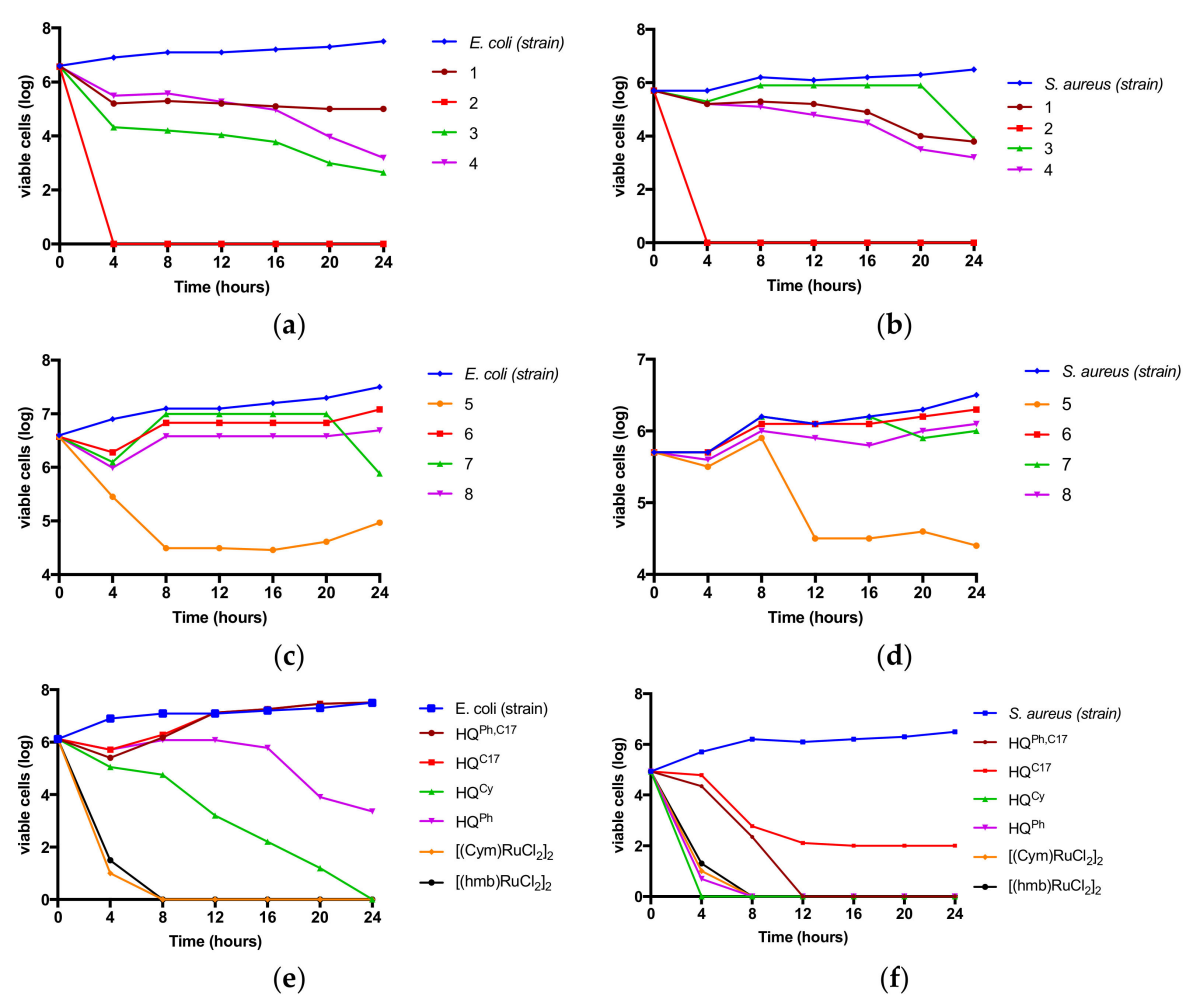
Time-kill kinetics assays with complexes 1–4 (**a**) and (**b**), complexes 5–8 (**c**) and (**d**), and proligands HQ^R^ and the metal precursors [(cym)RuCl_2_]_2_ and [(hmb)RuCl_2_]_2_ (**e**) and (**f**).

A time-kill kinetics assay was used to analyze the antibacterial activity of (arene)Ru(II) complexes 1–8 and proligands HQ^R^ against *E. coli* and *S. aureus*, with the growth inhibition evaluated in terms of log-reduction. The results are presented by grouping complexes 1–4, complexes 5–8 and the proligands separately (Figure 1). Among complexes 1–4, characterized by the presence of acylpyrazolones with a long aliphatic chain in either the 3 or 4 position of the pyrazolone ring, complex 2, with hmb and Q^Ph,C17^ ligands bound to the Ru(II) ion, showed the highest efficacy against both Gram- *E. coli* and Gram+ *S. aureus*, achieving a complete inhibition of *E. coli* and *S. aureus* growth just after 4 h (Figure 1a,b). This antibacterial activity is the highest among all complexes (1–8), and corresponds to a log-reduction higher than 3 log units, which implies strong antibacterial activity of complex 2. Complex 3, bearing cymene and Q^C17^ ligands on the Ru(II) fragment, also showed a good efficacy, in particular against *E. coli*. Significant antibacterial activity (1–3 log reduction) was shown by complex 3 after 4 h, achieving a log reduction higher than 3 after 16 h, corresponding to a strong activity. However, complex 3 showed a significant reduction against *S. aureus* only after 24 h. The growth of *E. coli*, in the presence of complex 3, resulted on a log reduction of 4.90, which represents a strong antibacterial activity. Complex 4, with hmb and Q^C17^ ligands coordinated to the Ru(II) ion, showed the same trend against both *E. coli* and *S. aureus*, with a significant activity at 16 h and a strong activity after 20 h. Complex 1 showed the lowest activity among 1–4, achieving only a significant activity after 24 h.

Complexes 5–8, that contain Q^R^ ligands bearing a phenyl or a cyclohexyl group on the acyl moiety, were also evaluated with complex 5 showing the highest activity against both *E. coli* and *S. aureus*, i.e., exhibiting a significant activity at 8 h against *E. coli* and at 24 h against *S. aureus*. Complex 7 displays a significant activity (1–3 log reduction) against *S. aureus*. The other complexes (6 and 8) showed negligible antibacterial activity (Figure 1c,d).

Proligands HQ^R^ showed a different effectiveness towards *E. coli* and *S. aureus* relative to the complexes. They all showed a strong antibacterial activity (>3 log unit reduction) against *S. aureus* after 8 h (Figure 1f). In contrast, *E. coli* inhibition was only observed for proligands HQ^Cy^ and HQ^Ph^, with some differences. Proligands HQ^C17^ and HQ^Ph,C17^ exerted a significant activity after 4 h, after which their efficacy decreased with time (Figure 1e). Strong antibacterial activity was displayed by proligands HQ^Cy^ and HQ^Ph^ after only 4 h, with an increase in efficacy over a period of 24 h. To obtain some insights on the specific biological contribution of the organometallic precursors employed in 1–8, [(cym)RuCl_2_]_2_ and [(hmb)RuCl_2_]_2_ were tested against *E. coli* and *S. aureus* (Figure 1e,f), and were found to display very strong antibacterial activity after 4 h. Note that these precursors are highly reactive and are not suitable for immobilization in polystyrene. Concerning the potential mechanism for the antibacterial activity of our ruthenium complexes, we can related to the well-known chelation theory and, additionally, also liposolubility mainly in the case of complexes 1–4, which enhances their penetration into lipid membranes of the bacterial cell walls. This in turn will interrupt the respiratory processes of the cell, blocking the synthesis of proteins and growth of the bacteria [41,42,43]. 

### 2.4. Amphipathic Behavior of Complexes 1–4

By dissolving complex 1 (2 mg) in MeOH (10 mL) and then adding H_2_O (10 mL), we observed the formation of a dispersion that is not retained when filtered with 1–3 μm porosity filter paper. Therefore, the formation of micelles smaller than 1 μm was hypothesized, due to the presence of the C_17_ aliphatic chain in the R_2_ position of the acylpyrazolone ligand, endowing the complex with surfactant-like properties, i.e., with the metal centre as the polar head and the long aliphatic chain representing the apolar moiety. As expected, the same amphipathic behaver was also observed with complexes 2–4, which in common with 1 have a long aliphatic chain in their structure.

**Figure 2:**
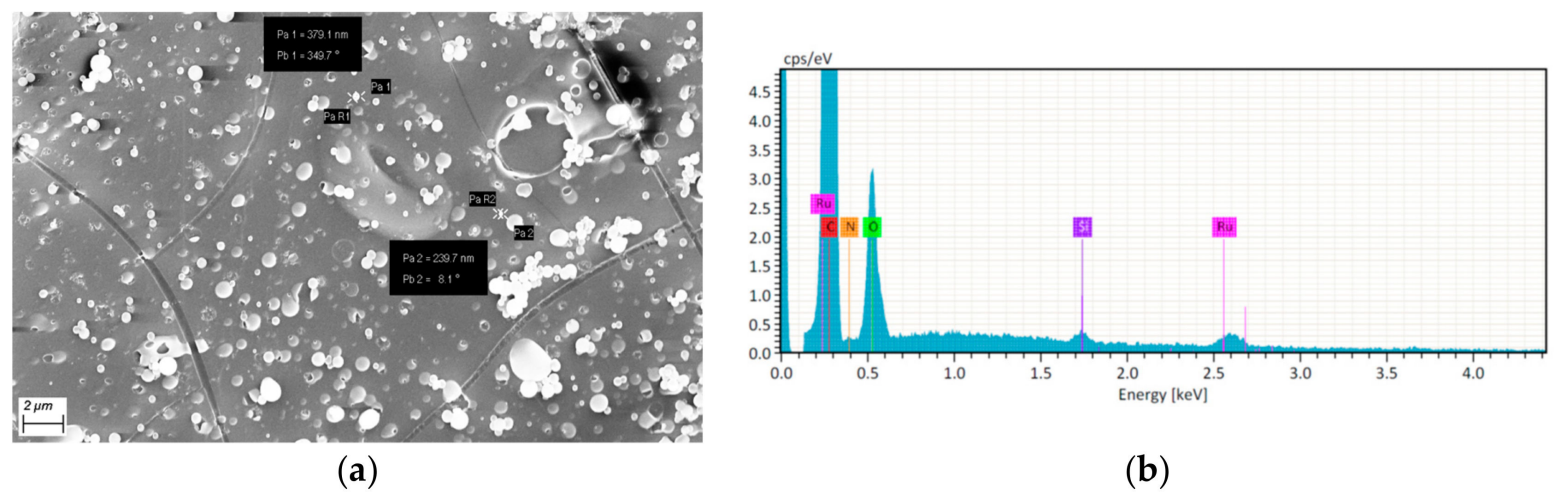
(**a**) SEM image of sample A obtained by deposition of 1 after evaporation of a micellar dispersion of 1 in MeOH/H_2_O onto a polystyrene support, and (**b**) EDX spectrum of the spherical aggregates.

To verify the micellar nature of 1 the complex was dispersed in MeOH/H_2_O on a polystyrene (PS) surface. The obtained material (sample A) was analysed by SEM and spherical aggregates with dimensions in the range 200 to 800 nm were observed (Figure 2a). The presence of complex 1 was confirmed by elemental analysis performed by Energy Dispersive X-ray (EDX), which highlights the presence of ruthenium (Figure 2b).

**Scheme 3:**
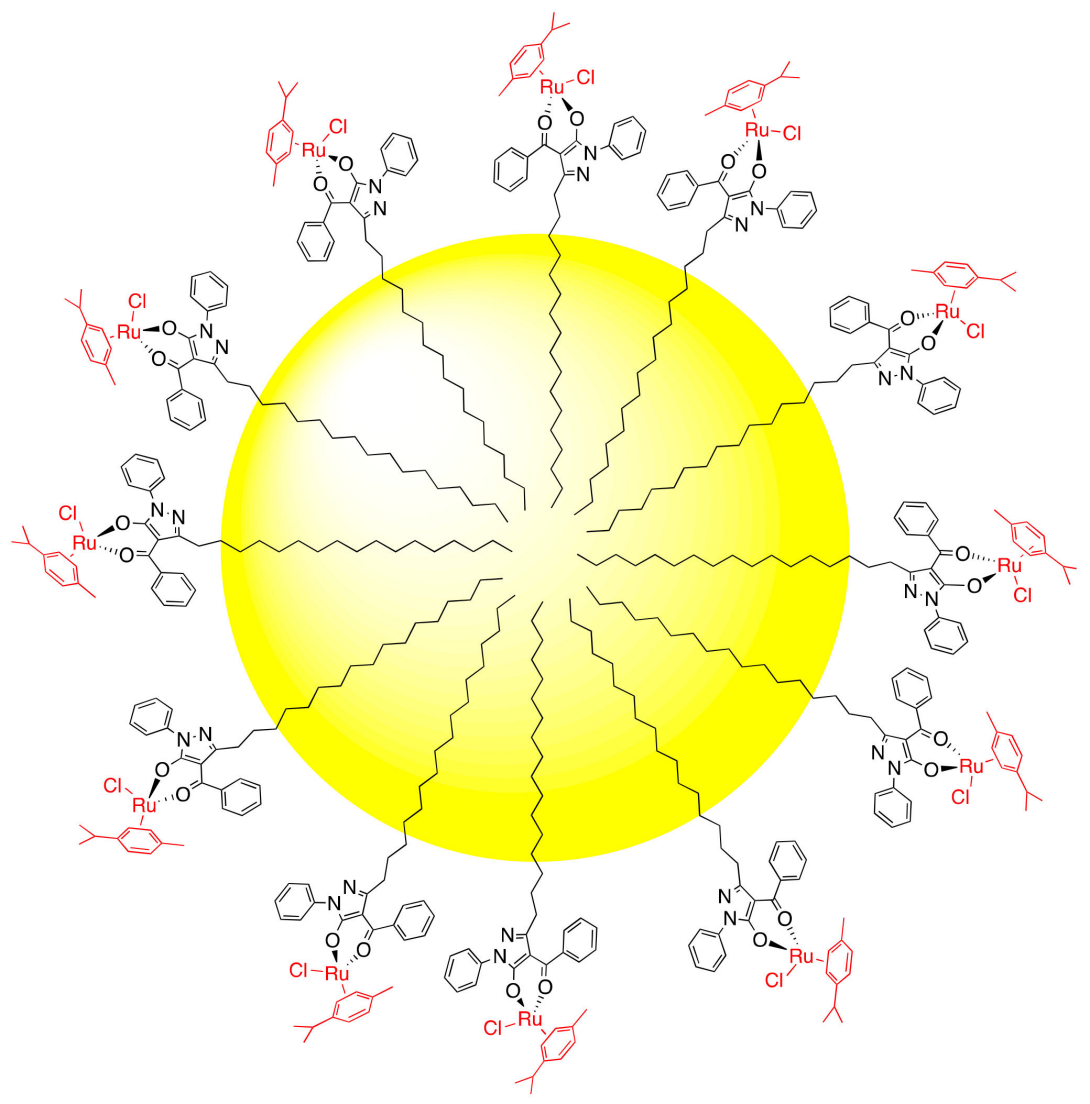
Tentative structure of the nanometric micellar assembly composed of complex 1.

The SEM image, recorded by detecting the signal of the backscattered electrons, demonstrates how ruthenium, the heaviest element present in the sample, is positioned on the surface of micelles. Atoms with larger Z values usually give more intense signals, highlighted here by lighter areas in the image (Figure 2a). We hypothesize that the micelles assemble with the (arene)Ru(II) fragment of complex 1 on the periphery and the long aliphatic chains directed toward the center due to hydrophobic interactions, as depicted in Scheme 3. 

**Figure 3:**
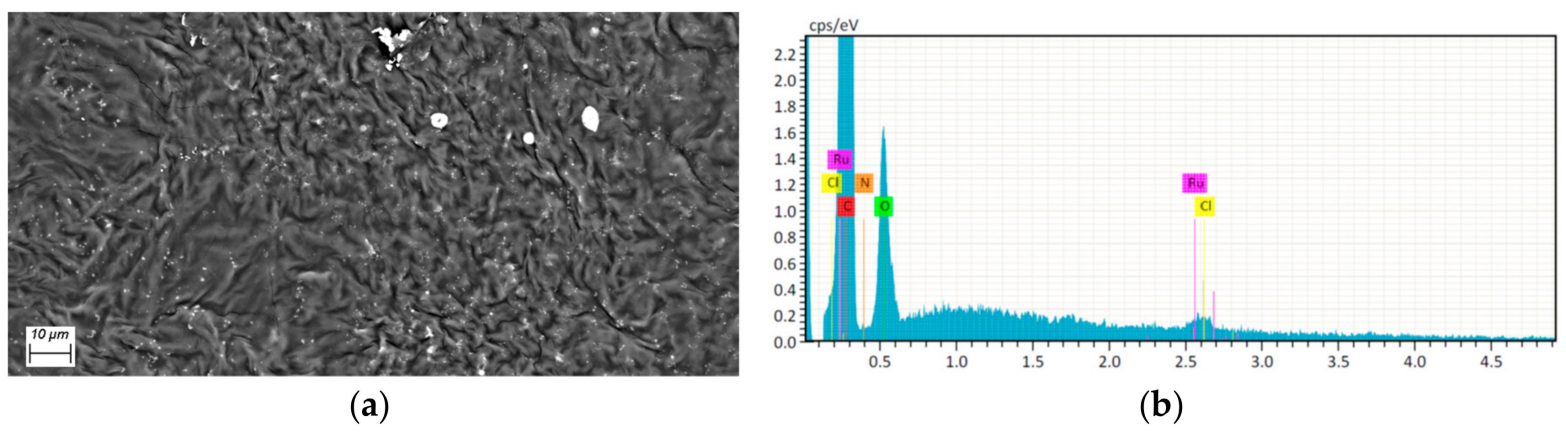
(**a**) SEM image of sample B obtained by direct addition of complex 1 to fused low-density polyethylene (LDPE) and (**b**) EDX spectrum of the aggregates.

Another material (sample B) was prepared by adding complex 1 directly to fused low-density polyethylene (LDPE) in 1:1000 w/w ratio, at a temperature of 110 °C, and then cooling the mixture to give a film with thickness 1 mm. SEM analysis reveals aggregates of dimensions from 1 to 4 μm spread over the LDPE surface (Figure 3).

**Scheme 4:**
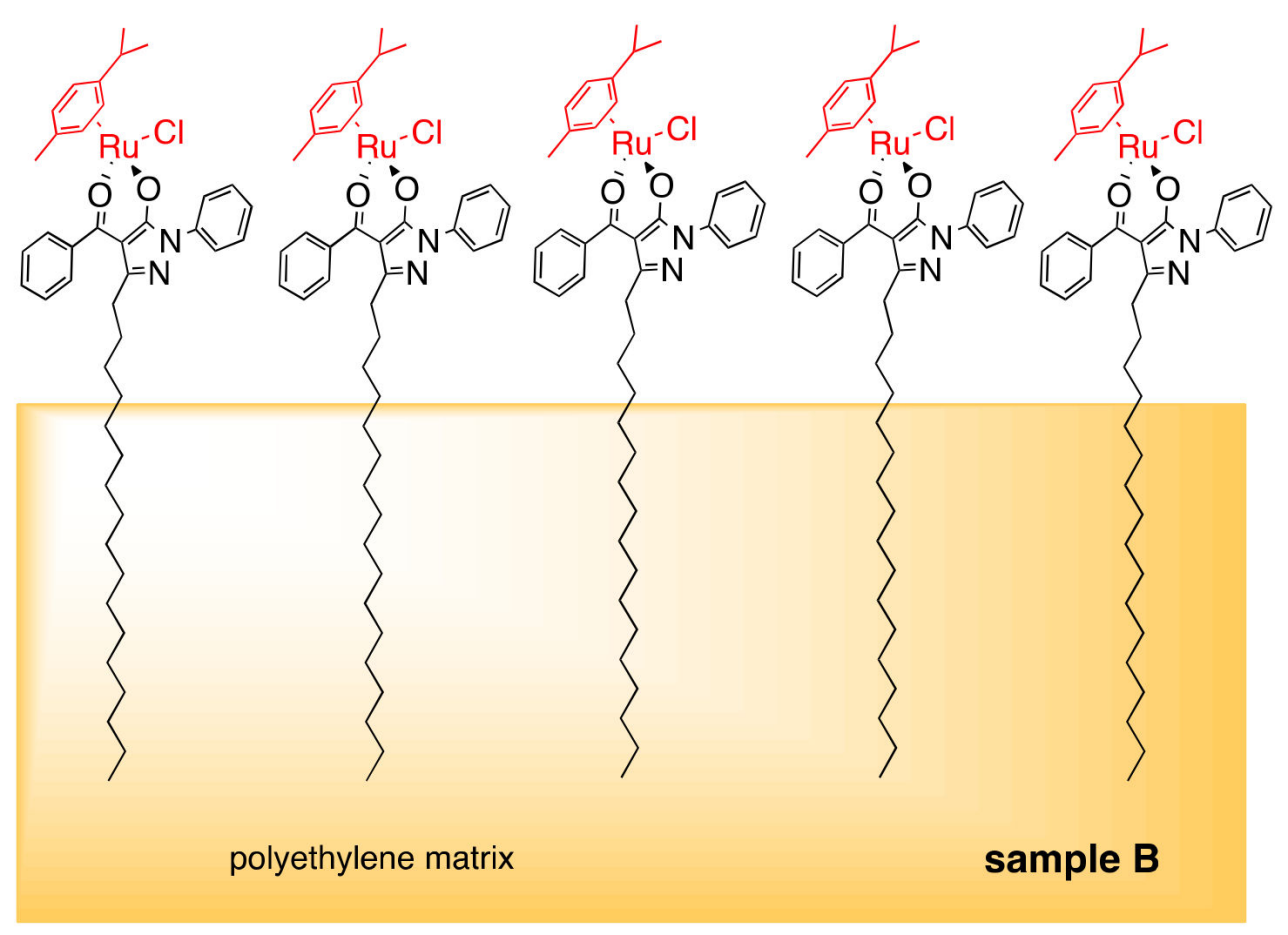
Tentative structure of complex 1 immobilized on the surface of LDPE in sample B formed via hydrophobic interactions of long aliphatic chains with bulk polyethylene.

The observed structure is possibly due to end-tethering of complex 1 with the (arene)Ru(II) fragment oriented away from the LDPE surface and the apolar part of the aliphatic chain adhered to the LDPE surface through hydrophobic interactions (Scheme 4).

### 2.5. Preparation of Samples PS1–PS4

A common procedure to tether metal complexes on a PS surface is based on functionalization of the PS with organic fragments containing suitable donor atoms able to covalently interact and immobilize a metal-containing moiety, e.g., an amine moiety on a polystyrene-modified surface was used to immobilize Pd(II) acyclic diaminocarbene complexes [44,45]. With the aim of firmly tethering the long aliphatic chain present in complexes 1–4 to the PS surface through a simpler, faster and more economic non-covalent immobilization procedure, we used acetone as solvent capable to solubilize both PS and complexes 1–4. Thereby, a drop of acetone solution containing the Ru(II) complex (conc. 1.2 × 10^−4^ M) was deposited on the surface of a square PS sample with dimensions 20 mm × 20 mm and thickness 1.2 mm (Figure S8 in Supplementary ), in which the ruthenium complex and a solubilized thin polystyrene layer coexist, allowing the C_17_ aliphatic chain in 1–4 to interact with the PS chains through hydrophobic interactions. Subsequent slow evaporation of the acetone solution favors the tethering of Ru(II) complexes on the PS surface, in a similar way to that proposed in Scheme 4. To remove any residual (arene)Ru(II) complex from the surface of PS not immobilized, samples PS1–PS4 were washed with methanol (as complexes 1–4 are soluble in this solvent whereas PS is insoluble). Complexes 5–8, do not possess amphipathic behavior, due to the absence of the long aliphatic chain in its structure, differently to complexes 1–4. Only the latter are in fact able to tether to polystyrene surface by our procedure of immobilization. For this reason, complexes 5–8 were not further investigated. 

**Figure 4:**
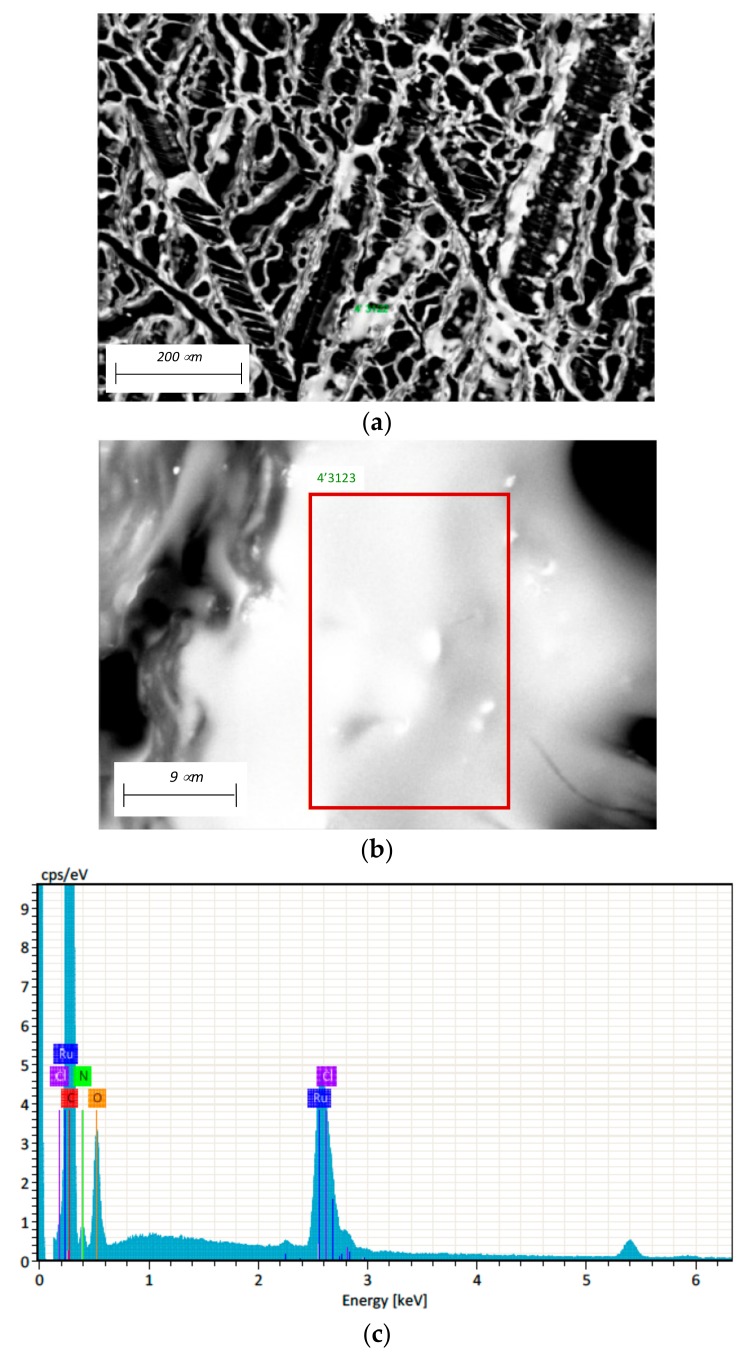
(**a**) Surface of PS2, (**b**) magnification of a light streak containing compound 2 and (**c**) EDX elemental analysis on the area framed by the red box in (**b**).

The surfaces of samples PS1–PS4 were characterized by SEM, showing a homogeneous distribution of the complexes. As an example, Figure 4 shows the surface morphology of the PS2 sample. The dark furrows and light streaks with dimensions of 10–50 μm are observed, the latter containing the ruthenium compound 2, as confirmed by the elemental analysis (Figure 4c and Table 1) on the area framed by the red box in Figure 4b, where a magnification of a light streak is shown.

**Figure 5:**
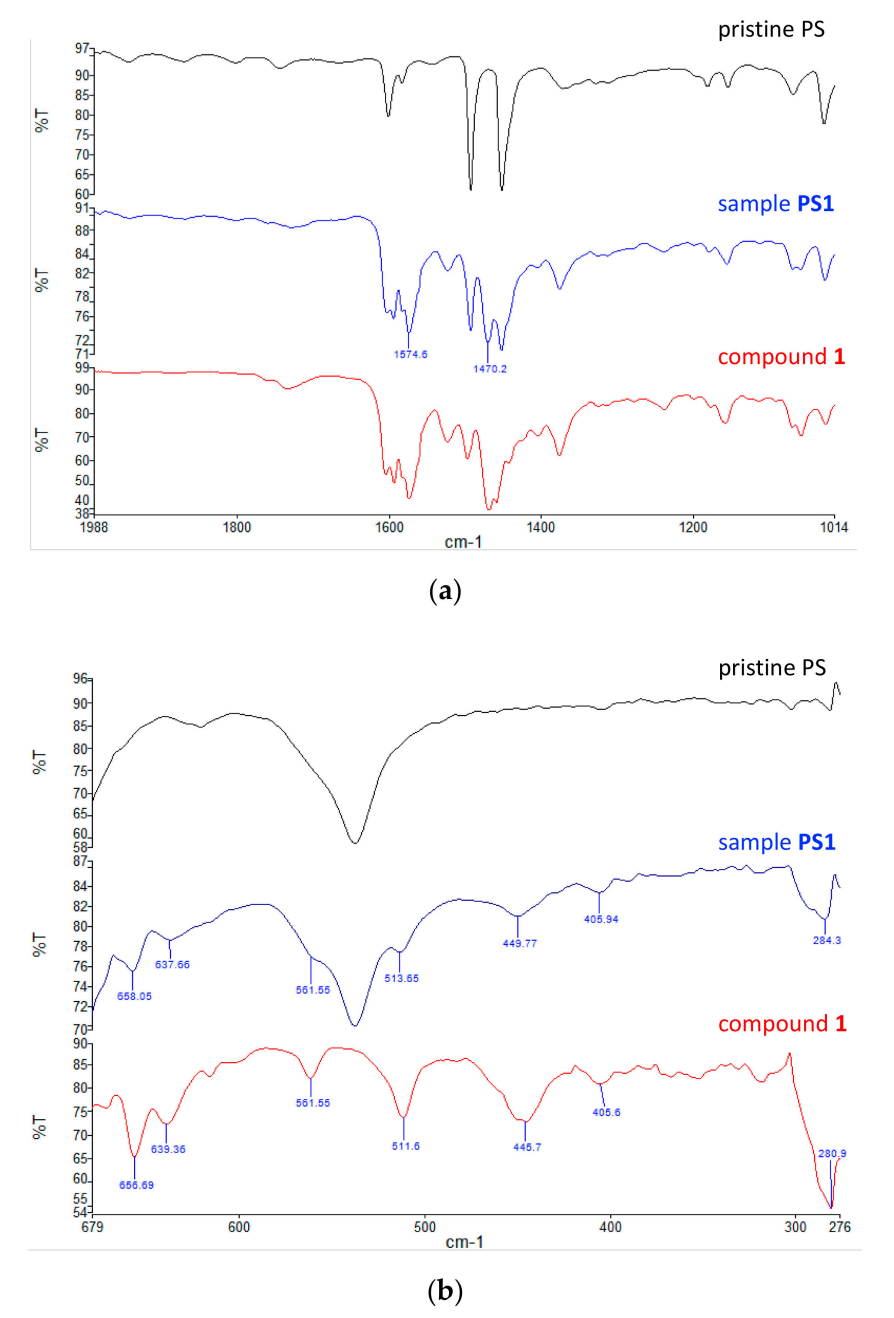
Comparison of the IR spectra of pristine PS, PS1 and compound 1 in (**a**) the medium region and (**b**) the far region.

Samples PS1–PS4 were also investigated by reflectance IR spectroscopy, with the absorptions due to the immobilized complexes being clearly identified, and essentially unchanged upon tethering (Figure 5).

### 2.6. Antibacterial Activity of PS1–PS4 According to ISO Standard

**Figure 6:**
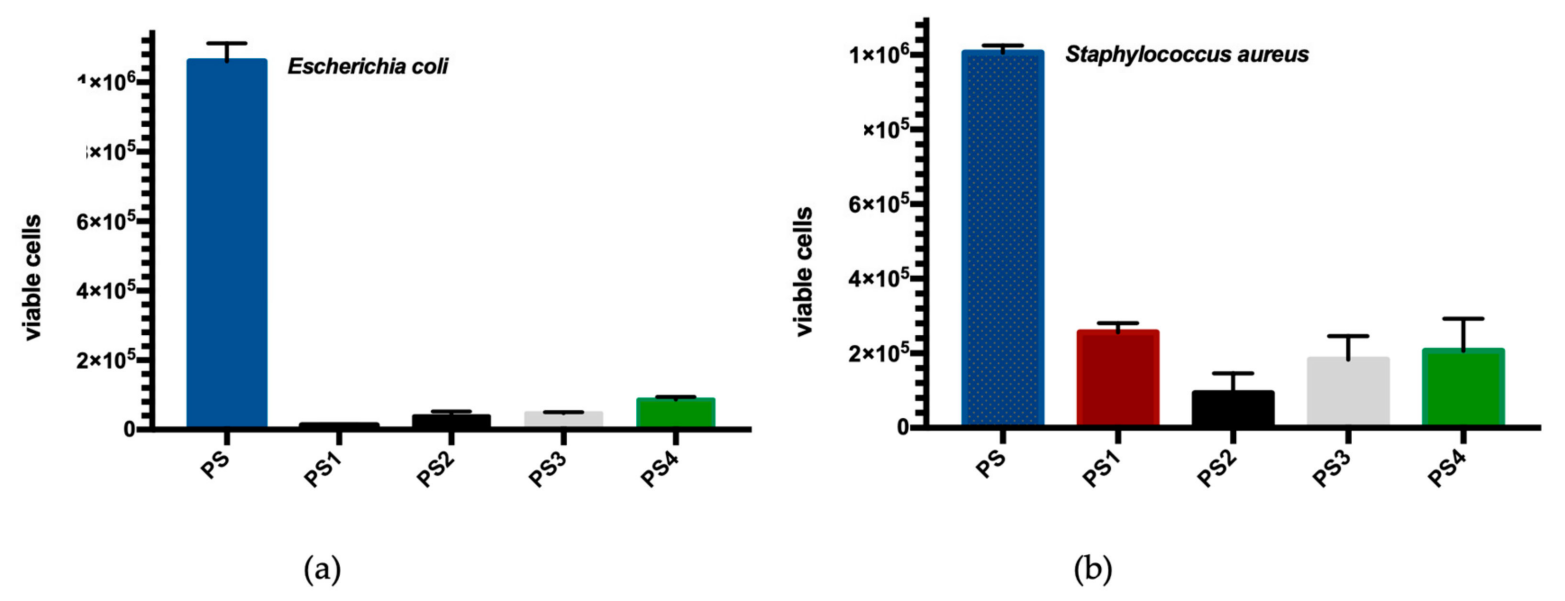
Antibacterial activity of PS1–PS4 against (**a**) *E. coli* and (**b**) *S. aureus* with respect to pristine PS, determined according to ISO standard and JIS [46,47].

To investigate the antibacterial activity of surfaces PS1–PS4 by contact, a standardized ISO test was carried out [46]. The results were elaborated according to the Japanese industrial standard (JIS) [47]. Figure 6 shows the results obtained against *E. coli* and *S. aureus* after 24 h of treatment, where the negative control is represented by pristine PS. PS1 showed the highest performance against *E. coli*, with a good antibacterial performance. 

Against *S. aureus*, instead, all samples showed a reduction of the antibacterial activity exhibiting the same behavior. The analysis of data highlighted that PS1, PS3 and PS4 achieved a Log reduction lower than 1, corresponding to a percentage of 68.4% to 90%. Only the PS2 showed a medium antimicrobial performance with a percentage reduction of 90% to 99%. Based on these trends, the influence of the acylpyrazolone ligand on the efficiency of ruthenium complexes 1–4 immobilized to the surface of PS, especially against *E. coli*, may be ascertained. 

**Scheme 5:**
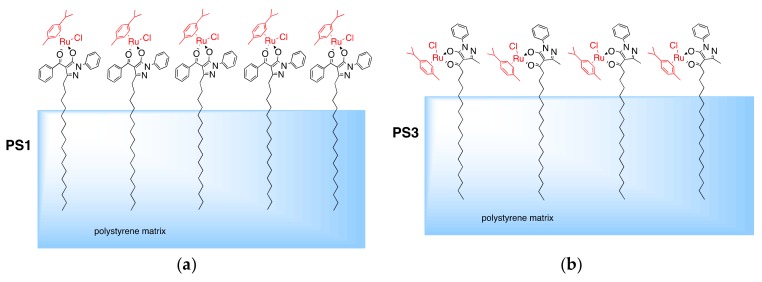
Different orientations of tethered molecules of 1 and 3 with respect to the PS surface in PS1 (**a**) and PS3 (**b**).

The aliphatic chain in the Q^Ph,C17^ ligand is pseudo-trans to the (arene)Ru(II) moiety, which could allow stronger tethering of the complexes to the PS surface and, hence, a good antibacterial activity through the arene-ruthenium moiety. In the Q^C17^ ligand, the aliphatic chain is pseudo-cis to the ruthenium moiety, which could somewhat reduce its ability to exert the antibacterial activity (Scheme 5).

## 3. Materials and Methods

Solvents were used as supplied or distilled using standard methods. All chemicals were purchased from Aldrich (Milwaukee, St. Louis, MO, USA) and used as received. The dimers [Ru(arene)Cl_2_]_2_ (arene = *p*-cymeme (cym) or hexamethylbenzene (hmb)) were purchased from Aldrich. The proligands HQ^R^ (R = Ph, Cy or C17) were synthesized using literature methods [34]. Complexes 3–8 were synthesized using a procedure previously reported [31,32].

IR spectra were recorded on a Perkin–Elmer Frontier FT-IR instrument (PerkinElmer, Inc., Waltham, MA, USA). ^1^H and ^13^C NMR spectra were recorded on a 500Bruker Ascend (500 MHz for ^1^H, 125 MHz for ^13^C) instrument (Bruker Corporation, Billerica, MA, USA) operating at room temperature relative to TMS. Positive ion electrospray mass spectra (ESI-MS) were obtained on a Series 1100 MSI detector HP spectrometer (Agilent Technologies, Santa Clara, CA, USA), using methanol/dichloromethane as solvent for complexes 1–8. Solutions (3 mg/mL) for electrospray ionization mass spectrometry (ESI-MS) were prepared using reagent-grade methanol. Melting points are uncorrected and were recorded on a STMP3 Stuart scientific instrument (Cole-Parmer, Stone, UK) TGA was performed on a PerkinElmer Pyris-1 thermogravimetric analyzer (PerkinElmer, Inc., Waltham, MA, USA), and the samples were heated from 30 to 850 °C at a heating rate of 10 °C min^−1^ under a nitrogen atmosphere flow (20 mL min^−1^). Samples for microanalysis were dried in vacuo to constant weight (20 °C, ca. 0.1 Torr) and analysed on a Fisons Instruments 1108 CHNS-O elemental analyzer (Fisons Instruments, Ipswich, UK).

### 3.1. Synthesis of Precursor 3-heptadecyl-1-phenyl-1H-pyrazol-5(4H)-one HP^Ph,C17^

Phenylhydrazine (1.267 g, 11.5 mmol) was added to a methanol solution (100 mL) of ethyl stearoylacetate (4.070 g, 11.5 mmol) in a 250 mL flask. The mixture was stirred at reflux for 12 h, the solvent removed on a rotary evaporator and a brown oily product washed with diethyl ether (20 mL). A solid red-brown powder slowly formed. After filtration, the red-brown powder was dried in vacuo to constant weight and shown to be 3-heptadecyl-1-phenyl-1H-pyrazol-5(4*H*)-one (4.309 g, 10.81 mmol, 94% yield). The compound is soluble in chlorinated solvents, acetone, DMF, diethyl ether and *n*-hexane diethyl ether and *n*-hexane, slightly soluble in hot ethanol and acetonitrile and insoluble in methanol and water. Mp: 64–65 °C. Anal. Calcd for C_26_H_42_N_2_O (MW 398.62): C, 78.34; H, 10.62; N, 7.03%. Found: C, 78.04; H, 10.60; N, 6.95%. IR (cm^−1^): 3049w, 2917vs, 2850s, 1615s, 1594m, 1566s, 1499s, 1470m, 1408m, 1355m, 1309m, 1151w, 1013w, 898w, 798m, 753vs, 718m, 688s, 664sh, 610m, 556w, 498m, 441w, 405w, 328w, 309m, 270w. ^1^H NMR (CDCl_3_, 298 K): δ 0.90t, 1.28br, 1.45br, 1.76br, 2.64br, 2.73t (35H, 3–(C*H*_2_)_16_C*H*_3_), 2.38s (2H, 4–C*H*_2_), 7.18t, 7.41t, 7.96d (5H, *H*_arom_). ^13^C{^1^H} NMR (CDCl_3_, 298 K): δ 14.1s, 22.7s, 29.3, 29.5br, 29.7br (*C*_17_H_35_ of HP^C17,Ph^), 32.0s (*C*4), 118.7, 119.3s, 119.6s, 128.8s, 128.9s, 120.0s, 129.1s (C_arom_ of HP^C17^).

### 3.2. Synthesis of Proligand (3-heptadecyl-5-hydroxy-1-phenyl-1H-pyrazol-4-yl)(phenyl)methanone HQ^Ph,C17^

3-Heptadecyl-1-phenyl-1H-pyrazol-5(4H)-one (1.0 g, 2.5 mmol) was placed in a flask equipped with a stirrer, separating funnel and a reflux condenser and dissolved in dry dioxane (60 mL) by warming. Calcium hydroxide (0.39 g, 5.02 mmol) and then benzoyl chloride (0.36 g, 2.5 mmol) were added, the latter dropwise over 10 min. The mixture was heated to reflux overnight and then poured into HCl 2 N (20 mL) to decompose the calcium complex. The resulting clear solution was heated to reduce the volume to one half, then dichloromethane (10 mL) was added and two phases formed. The organic phase, separated from the aqueous phase, was evaporated under reduced pressure to give a light brown solid, which was recrystallized from diethyl ether, dried to constant weight and shown to be 3-heptadecyl-5-hydroxy-1-phenyl-1H-pyrazol-4-yl)(phenyl)methanone (1.14 g, 2.27 mmol, 90% yield). The compound is soluble in chlorinated solvents, acetone, DMF, diethyl ether and *n*-hexane, slightly soluble in hot ethanol. Mp: 47–49 °C. IR (cm^-1^): 3031w, 2921vs, 2852s, 1599s, 1547vs, 1499s, 1458s, 1377m, 1304w, 1291w, 12189w, 1155w, 1069w, 1025w, 1001w, 949m, 907w, 836w, 755vs, 698vs, 689vs, 667w, 636s, 617w, 538m, 508m, 458w, 412m, 377w, 348w, 326w, 289m,, 270w. Anal. Calcd for C_33_H_46_N_2_O_2_ (MW 502.73): C, 78.84; H, 9.22; N, 5.57%. Found: C, 78.26; H, 9.18; N, 5.41%. ^1^H NMR (CDCl_3_, 298 K): δ 0.90t, 1.07br, 1.76br, 2.06br, 2.49t (35H, 3-(C*H*_2_)_16_C*H*_3_), 7.33t, 7.50t, 7.54t, 7.60m, 7.66d, 7.91d (10H, *H*_arom_). ^13^C{^1^H} NMR (CDCl_3_, 298 K): δ 14.1s, 22.7s, 28.5s, 29.0, 29.1s, 29.2s, 29.3s, 29.4s, 29.5s, 29.6s, 29.7s, 29.8, 31.9s, (*C*_17_H_35_ of HQ^C17,Ph^), 103.0s (*C*4), 120.1, 126.6s, 127.5s, 128.4s, 129.1s, 131.7s, 137.4s, 130.0s (*C*_arom_ of HQ^C17^), 152.3s (*C*3), 161.6s (C5), 192.2s (CO).

### 3.3. Synthesis of Complex [(Cym)Ru(Q^Ph,C17^)Cl] (1) 

To the proligand HQ^Ph,C17^ (166.0 mg, 0.33 mmol) dissolved in methanol (20 mL), KOH (18.5 mg, 0.33 mmol) was added. The mixture was stirred for 1 h at room temperature, and then [(cym)RuCl_2_]_2_ (100.0 mg, 0.165 mmol) was added. The resulting solution was stirred under reflux for 24 h. The solvent was removed under reduced pressure, and dichloromethane (10 mL) was added; the mixture was filtered to remove potassium chloride. The solution was left to evaporate at r.t., affording a brown-red viscous oil (224.3 mg, 0.29 mmol, yield 88%) that is soluble in methanol, ethanol, acetone, acetonitrile, DMSO, DMF, diethyl ether, n-hexane, and chlorinated solvents. Anal. Calcd for C_43_H_59_ClN_2_O_2_Ru (MW 772.5): C, 66.86; H, 7.70; N, 3.63%. Found: C, 67.20; H, 7.86; N, 3.75%. IR (cm^−1^): 3052w ν(C–H_arom_); 2921s 2851m ν(C–H_aliph_), 1604s, 1593s, 1573vs, 1523m ν(C=O); 1496m, 1468vs, 1375m, ν(C=C, C=N), 690s, 656s, 639m, 562w, 512m, 445m ν(Ru–O), 281s ν(Ru–Cl). ^1^H NMR (CDCl_3_, 298 K): δ 0.90t, 1.01m, 1.28m, 2.49t (35H, 3–(C*H*_2_)_16_C*H*_3_ of Q^Ph,C17^), 1.38d (6H, CH_3_–C_6_H_4_–CH(C*H*_3_)_2_), 2.18s (3H, C*H*_3_–C_6_H_4_–CH(CH_3_)_2_), 2.46m (1H, CH_3_–C_6_H_4_–C*H*(CH_3_)_2_), 5.32t, 5.35d, 5.49d, 5.50t (4H, CH_3_–C_6_*H*_4_–CH(CH_3_)_2_), 7.42t, 7.45t, 7.48t, 7.63t, 7.92d, 7.99d (10H, *H*_arom_ of Q^Ph,C17^). ^13^C NMR (CDCl3, 298 K): δ 18.0s (*C*H_3_–C_6_H_4_–CH(CH_3_)_2_), 22.3s, 22.4 (CH_3_–C_6_H_4_–CH(*C*H_3_)_2_), 14.4s, 18.9s, 22.4s, 22.1s, 22.7s, 28.3s, 28.5s, 29.1s, 29.2s, 29.3s, 29.4s, 29.5s, 29.6s, 29.7s, 31.9s, (*C*_17_H_35_ of Q^C17,Ph^), 30.8s (*C*H_3_–C_6_H_4_–*C*H(CH_3_)_2_), 79.0s, 79.1s, 80.5s, 81.3s, 82.3s, 82.4s, 96.6s, 99.4s (*C*H_3_–*C*_6_H_4_–CH(CH_3_)_2_), 105.7s (*C*4), 121.0s, 125.2s, 127.7s, 128.0s, 128.4s, 130.3s, 138.7s, 139.0s (*C*_arom_ of Q^C17^), 153.1s (*C*3), 163.7s (C5), 188.0s (CO). ESI-MS (+) (CH_3_OH) (*m*/*z*, relative intensity %): 737 [100] [(cym)Ru(Q^Ph,C17^)]^+^. TGA (mg% vs. °C): heating from 30 to 850 °C with a speed of 10 °C/min; from 180 to 850 °C progressive loss of due to decomposition of the complex, with a final residual of 9.0% weight. 

### 3.4. Synthesis of Complex [(Hmb)Ru(Q^Ph,C17^)Cl] (2)

Complex 2 was prepared similarly to 1 by using HQ^Ph,C17^ (166.0 mg, 0.33 mmol), KOH (18.5 mg, 0.33 mmol) and [(hmb)RuCl_2_]_2_ (110.0 mg, 0.165 mmol), but in this case a brown–red solid powder was isolated (240.4 mg, 0.30 mmol, yield 91%). The compound is soluble in acetone, CHCl_3_, CH_2_Cl_2_, DMF, diethyl ether, methanol, ethanol and slightly soluble in acetonitrile. M.p. 139–141 °C. Anal. Calcd for C_45_H_63_ClN_2_O_2_Ru (MW 800.5): 67.52; H, 7.93; N, 3.50%. Found: C, 67.31; H, 8.04; N, 3.45%. IR (cm^−1^): 3046w ν(C–H_arom_); 2921s, 2850m ν(C–H_aliph_), 1604s, 1593s, 1583vs, 1574vs, 1525m ν(C=O); 1495m, 1469vs, 1460m, 1373m, 1308w, ν(C=C, C=N), 690vs, 673w, 655s, 634w, 617w, 560m, 510m, 462m, 447s ν(Ru–O), 402w, 385 w, 333w, 287s, 280s ν(Ru−Cl). ^1^H NMR (CDCl_3_, 298 K): δ 0.90t, 1.01m, 1.28m, 2.49t (35H, 3–(C*H*_2_)_16_C*H*_3_ of Q^Ph,C17^), 2.05s, 2.11s (18H, C_6_(C*H*_3_)_6_), 7.42t, 7.49t, 7.53t, 7.66t, 7.91d, 8.01d (10H, *H*_arom_ of Q^Ph,C17^). ^13^C NMR (CDCl3, 298 K): δ 15.4s, 15.9s (C_6_(*C*H_3_)_6_), 14.1s, 22.7s, 28.3s, 29.1s, 29.2s, 29.3s, 29.4s, 29.5s, 29.6s, 29.7s, 31.9s, (*C*_17_H_35_ of Q^C17,Ph^), 89.6s, 89.7s (*C*_6_(CH_3_)_6_), 105.3s (*C*4), 120.8s, 124.8s, 127.6s, 127.9s, 128.3s, 130.1s, 138.9s, 139.8s (C_arom_ of Q^C17^), 152.8s (*C*3), 163.6s (C5), 187.9s (CO). ESI-MS (+) (CH_3_OH) (*m*/*z*, relative intensity %): 765 [100] [(hmb)Ru(Q^Ph,C17^)]^+^. TGA (mg% vs. °C): heating from 30 to 850 °C with a speed of 10 °C/min; from 150 to 850 °C progressive loss of due to decomposition of the complex, with a final residual of 9.0% weight.

### 3.5. Preparation of Samples PS1–PS4 

A thin film (2 drops) of an acetone solution of complex 1 (0.5 mg dissolved in 5 mL) was deposited on a square surface of polystyrene of dimensions 20 mm × 20 mm and 1.2 mm in thickness (Figure S8) and left to slowly evaporate at room temperature, affording a PS sample (sample PS1) in which the surface is coated with complex 1. Samples PS2–PS4 were prepared with the same procedure using complexes 2–4, respectively.

### 3.6. Antibacterial Activity of Proligands HQ^R^, of Complexes 1–8, and of Samples PS1–PS4

#### 3.6.1. Time-Kill Kinetics Assay

The antibacterial activity of proligands HQ^R^, and (arene)Ru(II) complexes 1–8 was tested against *Escherichia coli* ATCC 25922 (VWR International PBI s.r.l., Milano, Italy) and *S. aureus* ATCC 25923 (VWR International PBI s.r.l., Milano, Italy), respectively Gram-negative and Gram-positive. Bacterial inoculum (10^6^ CFU mL^−1^), was obtained by a series dilution of an aerobically overnight culture in Tryptone Soya Broth (OXOID), as the growth medium. For each bacterium, 50 μL of inoculum was added in the sterile test flask containing 5 mL of autoclaved physiological solution (0.85% NaCl). The bacteria concentration was also checked by recording the absorbance at 600 nm with a UV-1601 CE spectrophotometer (SHIMADZU CORPERATION) using Tryptone Soya Broth as blank. 20 mg of each powdered (arene)Ru(II) complex was then weighed and added to flask content. All flasks were kept on an IKA KS 130 BASIC platform shaker for speed of 160 rpm and 24 h. The concentration of 20 mg of ruthenium complex in 5 mL of culture was chosen because, based on preliminary tests, it was the best among three different concentrations (50 mg/5 mL, 20 mg/5 mL and 5 mg/5 mL) to obtain a good antibacterial activity with the lowest concentration of the antibacterial agent. To study the killing activity of proligands and complexes, periodic withdrawal of 5 μL at times 0, 4, 8, 12, 16, 20 and 24 h was collected for each sample. To obtain the bacterial colony count, the supernatant fraction was diluted and included uniformly into Petri dishes containing Plate Count Agar (OXOID). Adopting the same procedure, a sample strain was used as control, and the number of viable cells counted was reported on the logarithmic scale.

#### 3.6.2. Antibacterial Activity According to ISO Standard

The antibacterial activity by contact of samples PS1–PS4 was performed according to the ISO standard and the interpretation of the results elaborated according to the Japanese Industrial Standard (JIS) [46,47]. All tested square composites had dimensions of 20 mm × 20 mm (1.2 mm in thickness) and were tested, in triplicate, against two strains: (Gram-positive) *S. aureus* ATCC 25 923 and (Gram-negative) *E. coli* ATCC 25 922. Unloaded PS was used as the negative control. The appropriate culture medium was inoculated with the test microbes and cultivated for 24 h at 37 °C under aerobic conditions, to achieve the concentration of 10^6^ CFU/mL. Bacterial suspensions (0.4 mL) were inoculated onto the test surface and the inoculum was covered with a piece of low-density polyethylene film (40 × 40 mm), gently pressed down to spread the inoculum to the edges. The Petri dishes containing the inoculated test specimens were incubated at (35 ± 1) °C and a relative humidity of not less than 90% for 24 ± 1 h. After the incubation time, the inoculum was processed by adding 10 mL SCDLP broth (Soybean casein digest broth with lecithin and polyoxyethylene sorbitan monooleate). From SCDLP broth, tenfold serial dilutions were made in phosphate-buffered physiological saline (PBS-saline) and aliquots of 1 mL for each dilution were placed in Petri dishes, and 15 mL of plate count agar (PCA) was poured to disperse the bacteria. The inverted Petri dishes were incubated at (35 ± 1) °C for 48 h. After incubation, the numbers of colonies in the Petri dishes were counted. The numbers of bacteria surviving on the specimens tested were compared to the number of colonies present on the negative controls. The antibacterial activity (R) was determined according to the Japanese industrial standard [47] based on the following rating: no antimicrobial activity ≤ 0.5 log microbial growth reduction; slight antimicrobial activity = 0.5 − 1.0 log microbial growth reduction; significant antimicrobial activity > 1 to ≤3 log microbial growth reduction; strong antimicrobial activity > 3log microbial growth reduction.

## 4. Conclusions

We reported new (arene)Ru(II) complexes of acylpyrazolone proligands, some with a long aliphatic chain, and tested their antibacterial activity against Gram+ and Gram- bacterial colonies. The complexes display from medium to high activity, mainly those with the appended hydrophobic chain. These complexes were tethered to LDPE and PS surfaces through simple procedures affording composite materials endowed with efficient antibacterial activity.

## Figures and Tables

**Table 1:** EDX elemental analysis data on the area 4′3123 framed by the red box in Figure 3b.

Element	At. No.	Net	Mass [%]	Mass Norm. [%]	Atom	Abs. Error [%] (1 σ)	Rel. Error [%] (1 σ)
Carbon	6	329219	89.71	72.65	82.81	9.62	10.73
Nitrogen	7	4366	8.79	7.12	6.96	1.44	16.34
Oxygen	8	15301	11.79	9.55	8.17	1.59	13.45
Chlorine	17	24173	3.03	2.46	0.95	0.13	4.24
Ruthenium	44	52600	10.15	8.22	1.11	0.37	3.60
		**Sum**	**123.48**	**100.00**	**100.00**		

## Supplementary Materials

The following are available online at https://www.mdpi.com/1996-1944/13/3/526/s1: IR, ^1^H and ^13^C NMR spectra of proligand HQ^C17,Ph^, its precursor HP^C17,Ph^ and complexes 1 and 2.
