# Supplementary material for: Tethering (Arene)Ru(II) Acylpyrazolones Decorated with Long Aliphatic Chains to Polystyrene Surfaces Provides Potent Antibacterial Plastics
Source: Materials (Basel). 2020 Jan 22;13(3):526. doi: 10.3390/ma13030526 (PMC7040715; doi:10.3390/ma13030526)
Supplement: Supplementary file 1 [file materials-13-00526-s001.pdf]

Supplementary

Tethering (Arene)Ru(II) Acylpyrazolones Decorated with Long Aliphatic Chains to Polystyrene Surfaces Provides Potent Antibacterial Plastics

Corrado Di Nicola [1,*], Fabio Marchetti [1], Riccardo Pettinari [2], Alessia Tombesi [1], Claudio Pettinari [2], Iolanda Grappasonni [2], Paul J. Dyson [3] and Stefania Scuri [2,*]

1. School of Science and Technology, Chemistry Section, University of Camerino, Via S. Agostino 1, 62032 Camerino Macerata, Italy; fabio.marchetti@unicam.it (F.M.); alessia.tombesi@unicam.it (A.T.)
2. School of Pharmacy, Chemistry Section, University of Camerino, Via S. Agostino 1, 62032 Camerino Macerata, Italy; riccardo.pettinari@unicam.it (R.P.);claudio.pettinari@unicam.it (C.P.); iolanda.grappasonni@unicam.it (I.G.)
3. Institut des Sciences et Ingénierie Chimiques, École Polytechnique Fédérale de Lausanne (EPFL), 1015 Lausanne, Switzerland; paul.dyson@epfl.ch

* Correspondence: corrado.dinicola@unicam.it (C.D.N.); stefania.scuri@unicam.it (S.S.); Tel.: +39-0737-402338 (C.D.N.); +39-0737-402020 (S.S.)

Received: 5 December 2019; Accepted: 19 January 2020; Published: date

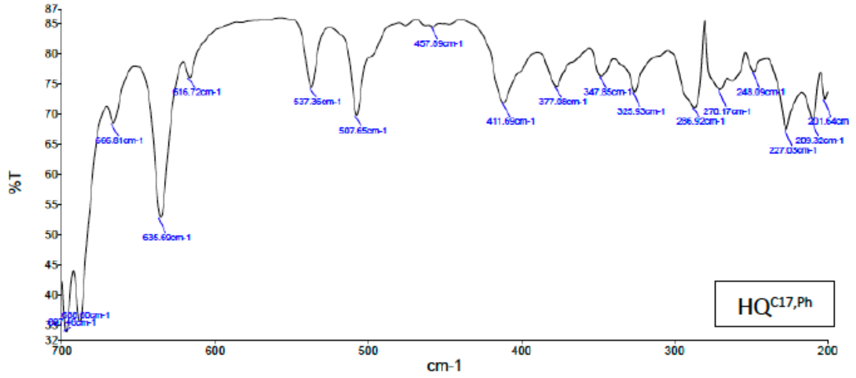

(a)

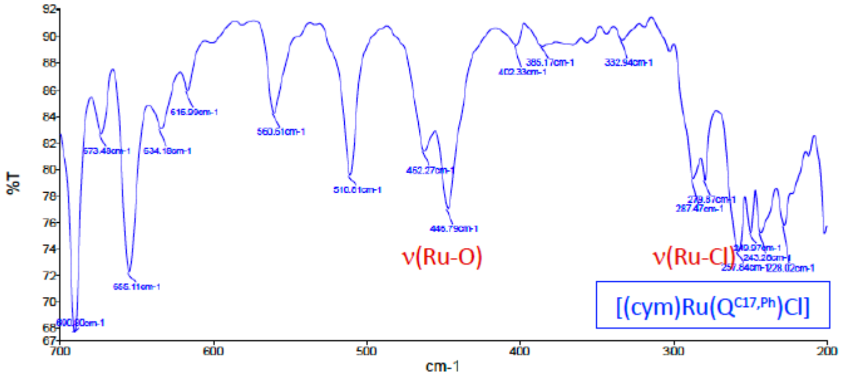

(b)

Figure S1. Far-IR spectra of (a) free proligand $HQ^{C17,Ph}$ and (b) complex **1**.

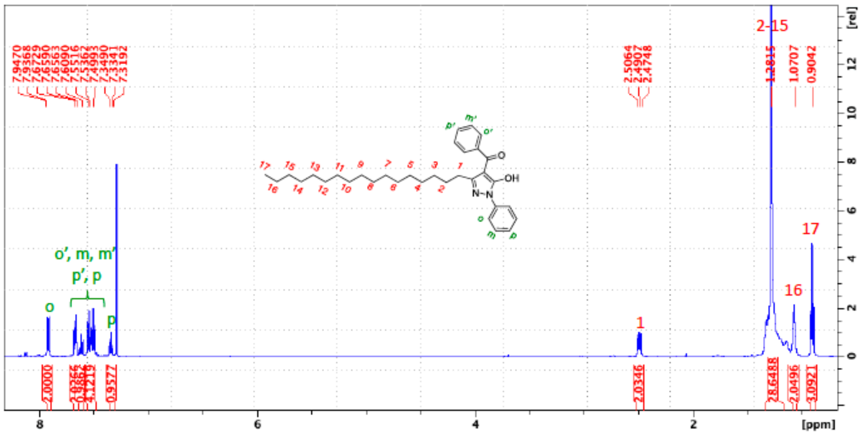

Figure S2. ^{1}H NMR spectrum of proligand $HQ^{C17,Ph}$ in $CDCl_3$ solution at 298 K.

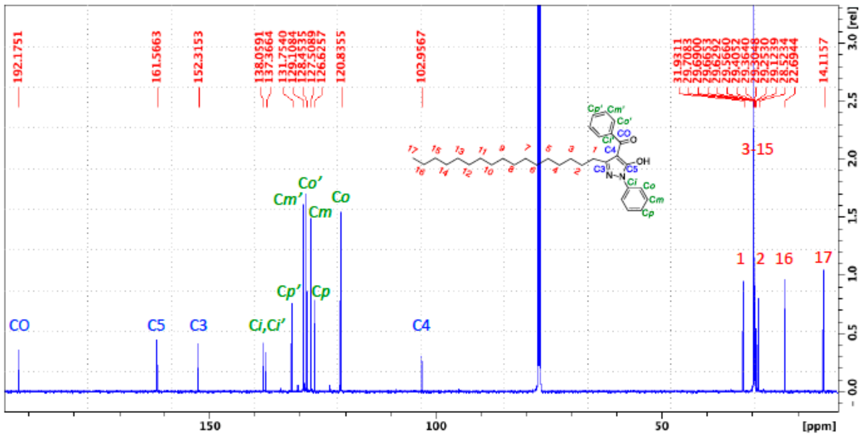

Figure S3. ^{13}C NMR spectrum of proligand $HQ^{C17,Ph}$ in $CDCl_3$ solution at 298 K.

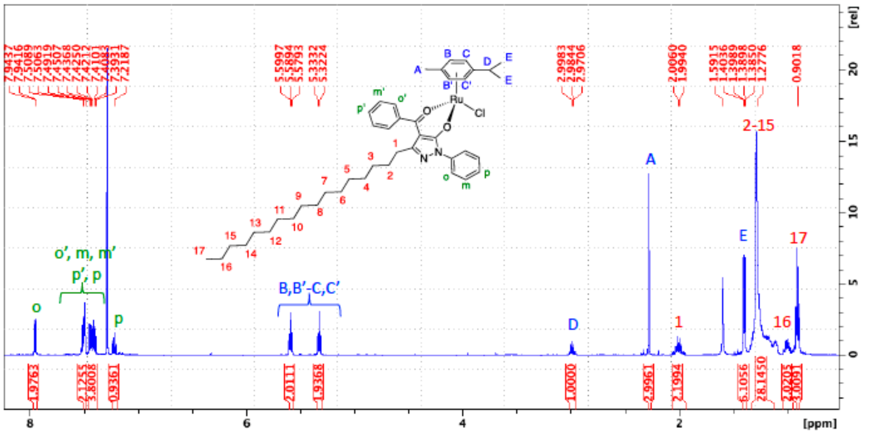

Figure S4. ^{1}H NMR spectrum of complex **1** in $CDCl_3$ solution at 298 K.

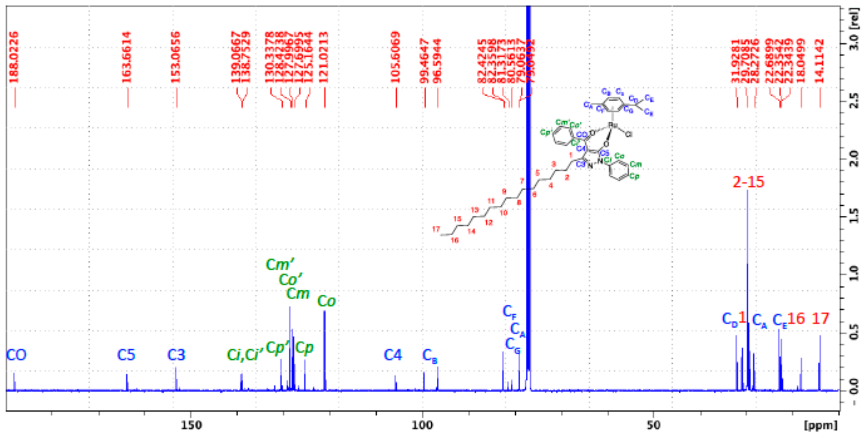

Figure S5. ^{13}C NMR spectrum of complex **1** in $CDCl_3$ solution at 298 K.

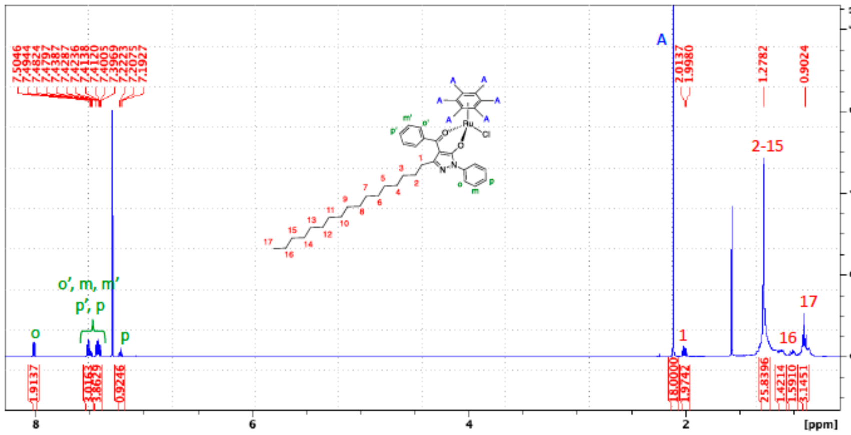

Figure S6. ^{1}H NMR spectrum of complex **2** in $CDCl_3$ solution at 298 K.

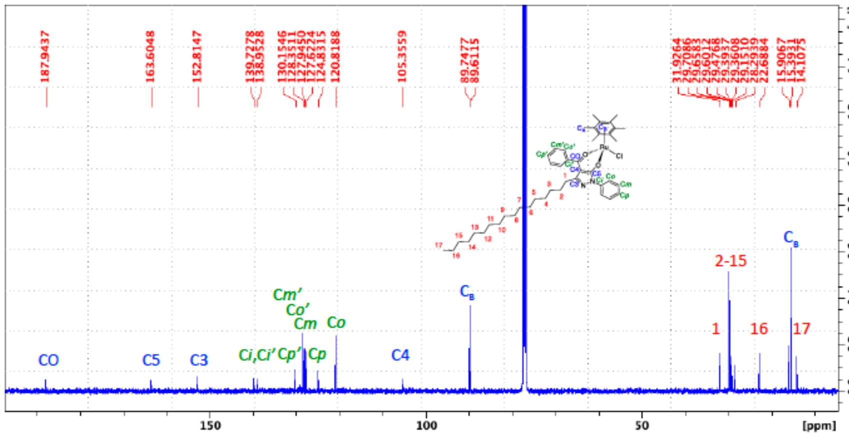

Figure S7. ^{1}H NMR spectrum of complex **2** in $CDCl_3$ solution at 298 K.

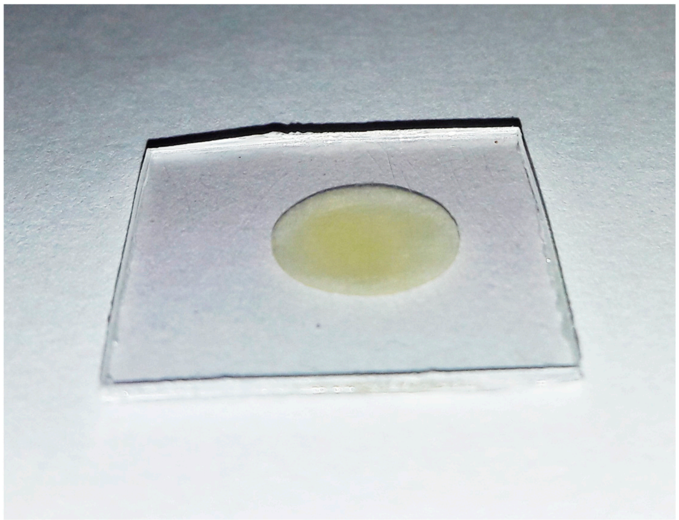
Figure S8. Polystyrene square sample with dimensions 20 x 20 mm and thickness 1.2 mm where a drop of acetone solution of **1** was deposited.
